# A case of complicated chronic rhinosinusitis with nasal polyps treated with dupilumab, in the UK: a case report

**DOI:** 10.1186/s13256-026-06122-8

**Published:** 2026-11-13

**Authors:** Emily Murdoch, Lucy Leeman

**Affiliations:** 1 University Hospitals Plymouth NHS trust, Plymouth, UK; 2 University of Plymouth, Plymouth, UK

**Keywords:** Dupilumab, Nasal polyps, Refractory, Exceptional, Inflammation, Funding

## Abstract

**Background:**

Chronic rhinosinusitis (CRS) is a prevalent inflammatory disorder of the nasal and paranasal mucosa, affecting approximately 1 in 10 people and contributing to substantial symptom burden and impaired quality of life. Phenotypically, CRS is classified into two subtypes based on the presence or absence of nasal polyps—benign inflammatory outgrowths of the nasal mucosa—which occur in roughly 20% of affected individuals. Chronic Rhinosinusitis with Nasal Polyps (CRSwNP) is predominantly driven by type 2 (T-helper 2) inflammation, typically characterised by eosinophilic infiltration and involvement of interleukins, such as IL-4, IL-5, and IL-13. Given this immunopathology, biologic therapies, such as Dupilumab, Omalizumab and Mepolizumab, initially developed for asthma, have shown efficacy in this patient population. They are readily used in the United States (US) and European Union (EU) in cases of severe CRSwNP, but are not currently authorised for NHS use.

**Case presentation:**

We describe a case of severe, refractory CRSwNP complicated by recurrent left periorbital abscess and dehiscence of the floor of the left frontal sinus in a white, male patient in his sixties. The condition persisted despite multiple surgical polypectomies, repeated courses of systemic antibiotics (oral and intravenous), topical and systemic steroids, and drainage of periorbital collections. At the time, Dupilumab was not routinely commissioned by the NHS for this indication. However, following a successful application to NHS England’s Individual Funding Request (IFR) process, funding for Dupilumab was granted.

**Conclusions:**

Treatment with Dupilumab led to complete resolution of symptoms and significant improvement in quality of life. The patient remains under regular follow-up with ongoing clinical stability and no reported adverse effects.

## Background

Chronic rhinosinusitis with nasal polyps (CRSwNP), a subtype of Chronic Rhinosinusitis, is a heterogeneous inflammatory condition with varying degrees of severity. First-line management typically includes intranasal corticosteroids, nasal saline irrigation, and intermittent short courses of systemic corticosteroids. In severe or refractory cases, surgical techniques are often necessary to remove polyp tissue and relieve symptoms [1]. Although most patients respond well to current treatments and do not require revision surgery, a subset of individuals with severe, refractory CRSwNP continue to experience disease progression despite maximal medical and surgical therapy. This subgroup of patients represents a significant therapeutic challenge, highlighting the importance of novel treatments and consideration of targeted biologic therapies [2].

This article explores a patient with recurrent CRSwNP complicated by threat to vision, and a severe detriment to quality of life.

## Case presentation

A previously well, white, male in his sixties developed CRSwNP (Table 1) and flexible nasendoscopy, performed by ENT, revealed bilateral grade 3 nasal polyps. Initial management included standard medical therapy as per ENT guidelines. Imaging also identified a frontal sinus osteoma, and the patient was subsequently listed for functional endoscopic sinus surgery (FESS).

**Table 1:** Social and past medical history of the patient

Social and past medical history of patient
Likely asthma
Inhalers, including salamol and fostair
No seasonal allergic rhinitis
Never smoked
No industrial exposure
No bird keeping

Although the initial surgical intervention was successful, the patient re-presented with vertical binocular diplopia, hyposmia, and hypogeusia. Imaging confirmed a left superior orbital abscess with frontal sinus dehiscence. He underwent urgent surgical drainage under joint ENT and ophthalmology teams.

Approximately 1 month later, the patient experienced recurrence of the same symptoms and Computerise Tomography (CT) imaging again confirmed a left orbital collection. He returned to theatre for revision drainage and was discharged with an extended course of antibiotics. Histopathological analysis revealed evidence consistent with inflammatory nasal polyps with the absence of fungal hyphae (Table 2).

**Table 2:** Microbiology, biochemistry and immunology investigations

Microbiology, biochemistry and immunology investigations
Mucin
Neutrophils present
No fungal hyphae
Presence of eosinophils
No bacterial growth
Skin prick testing to aeroallergens
Negative
Total IgE 148 kU/L (mildly elevated)
Blood cultures negative
CRP: <1–8 mg/L (over course of events)

Aeroallergens tested included dog and cat dander, mixed trees, feathers, mixed grass and timothy grass, house dust mite and mould

Despite prior interventions, the patient presented a third time with similar symptoms and underwent another combined surgical procedure. Post-operatively, he was managed with a tapering course of oral corticosteroids, antibiotics and antihistamines.

Given the recurrent nature of his CRSwNP within months and severe complications posing threat to his eyesight, he was referred to clinical immunology for further management and consideration of biologics. At the time of presentation, Dupilumab was not NICE (National Institute for health and Care Excellence) funded for treating CRSwNP. Following an extensive but successful funding application, treatment with Dupilumab was started. Initial doses were observed in hospital and then education given to continue the treatment at home. SNOT-22 (Sino-Nasal Outcome Test) scores were completed at baseline, and throughout treatment, to assess symptom burden. Follow-up with ENT 2 weeks after initiating Dupilumab demonstrated symptomatic improvement, with complete resolution of nasal polyps observed on flexible nasoendoscopy at 6 weeks. He has since been discharged from ENT follow-up and remains stable under immunology care with ongoing Dupilumab therapy, with no further recurrences or adverse effects noted. An annual report is provided to the NHSE IFR (National Health Service England Individual Funding Request) panel detailing patient progress and response to treatment. Included within this are any ongoing healthcare cost savings, such as reduced out-patient visits compared to pre-treatment levels, and improvements in symptom and quality of life scores compared to baseline. If continued benefit can be adequately demonstrated via this report, funding for a further year of treatment is authorised. A trial of extended dose interval has thus far been unsuccessful; however, a further trial may be considered in the future.

This case is exceptional for the following reasons;

- Extreme rapidity of recurrence of polyps and collections
- Absence of fungal hyphae
- Threat to vision and disfigurement
- Frontal sinus dehiscence

The severity of symptoms had a profound impact on the patient’s quality of life, with escalating concerns about potential unilateral vision loss and disfigurement. Encouragingly, the patient experienced rapid and sustained relief following initiation of therapy, contrastingly with previous surgical interventions, where symptoms would often recur within days. The current regimen involves self-administered subcutaneous injections every 2 weeks, which the patient is managing well. Interval dose duration extension has been attempted, however; there remains some anxiety around the potential return of symptoms. Notably, the patient reports a sensation of periocular tightness as they approach the end of the fortnightly cycle, which contributes to their hesitation in altering the dosing schedule.

**Fig. 1:**
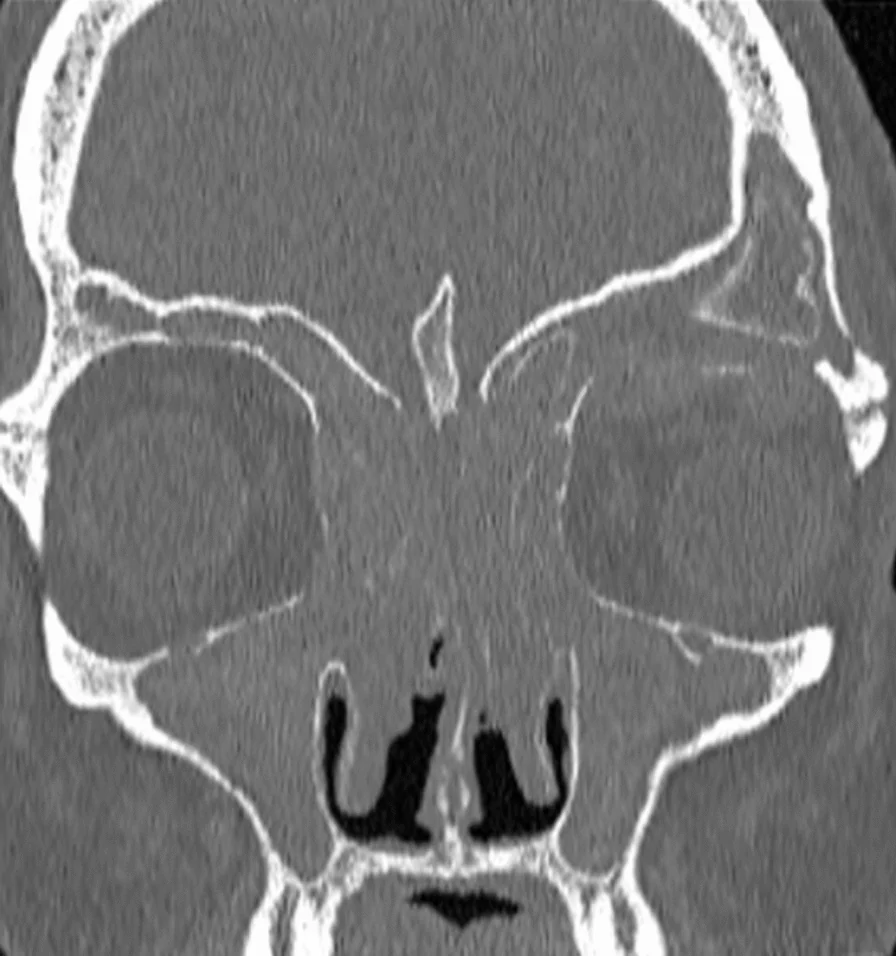
Coronal CT view

**Fig. 2:**
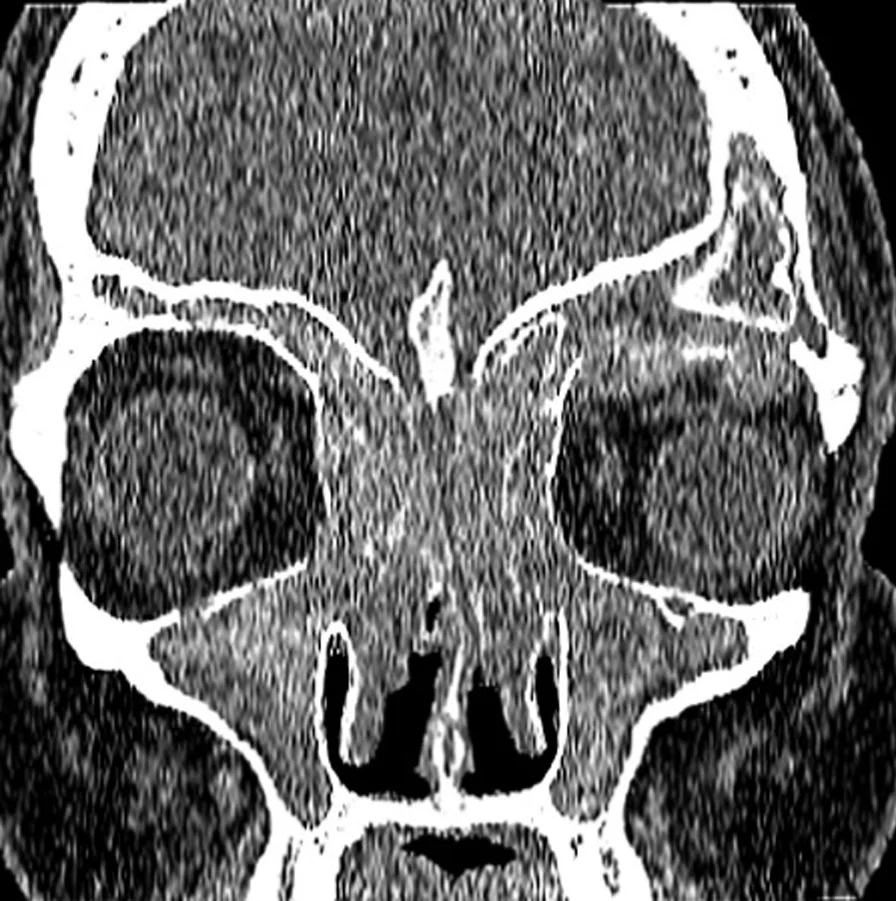
CT of a coronal soft tissue view

CT of the paranasal sinuses in the coronal plane, with bone reconstructions (Fig. 1) and soft tissue window (Fig. 2) shows complete opacification of the maxillary, ethmoid and frontal sinuses as well as extensive nasal polyposis bilaterally. There is erosion of the floor of the left frontal sinus resulting in extension of inflammatory soft tissue into the left orbit.

**Fig. 3:**
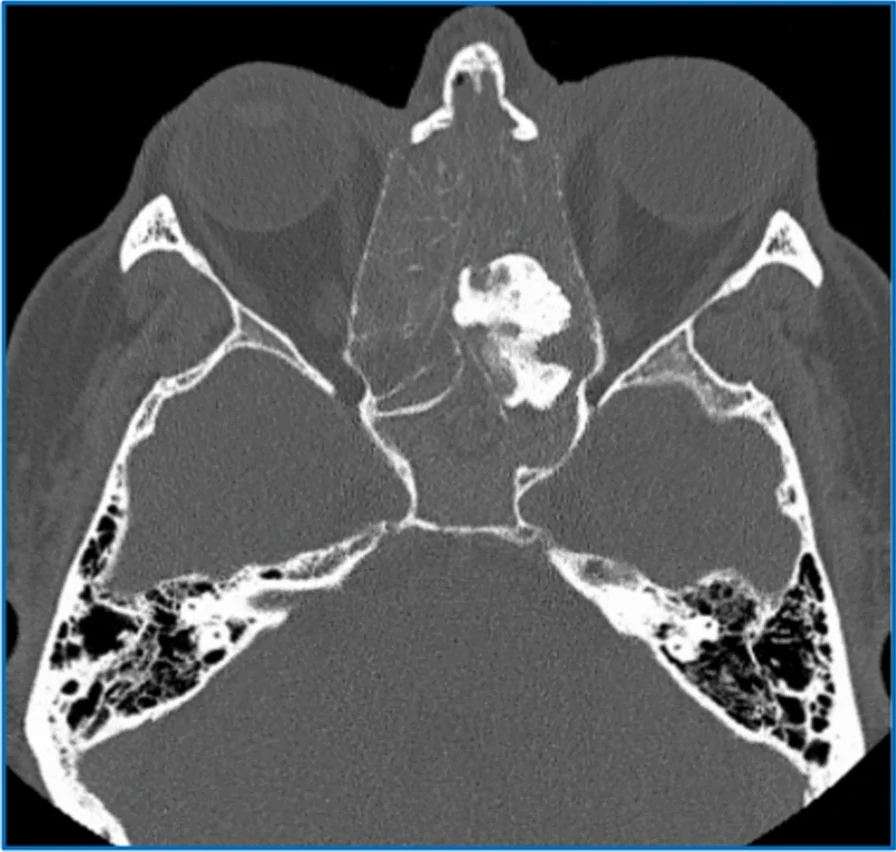
Axial view

CT of the paranasal sinuses axial view in bone window (Fig. 3) shows complete opacification of the ethmoid and sphenoid sinuses together with a left posterior ethmoid osteoma.

## Discussion

Chronic Rhinosinusitis is a diverse pathology which is acknowledged as two main phenotypes: one with nasal polyps and one without. However, phenotypic overlap is becoming more evident [1]. Up to a quarter of patients with CRS have nasal polyps and there are varying levels of treatments that are currently successful. These include medical therapies, including nasal steroids and saline irrigations. Some patients require oral steroids and antibiotics, however, if unsuccessful then surgical intervention is advised. Functional endoscopic sinus surgery (FESS) is the most common procedure performed by ENT surgeons to remove the polyps and clear the nasal passage of obstruction [2]. CRSwNP associated with type 2 inflammation is linked to an increased risk of nasal polyps’ recurrence [1].

Recurrence following surgery is observed in patients with elevated levels of interleukin-5 (IL-5) and tissue eosinophilia—both markers of type 2 inflammation [2]. IL-5 plays a central role in eosinophil differentiation, migration, activation, and survival, and its presence is strongly associated with disease recurrence and persistent symptom burden [1]. Similarly, interleukin-13 (IL-13), another type 2 inflammatory cytokine, contributes to airway inflammation and modulates nitric oxide (NO), an important mediator involved in respiratory tract immunity and inflammation [3]. IL-4 is imperative in class switching B cells into IgE antibodies. IgE bound to mast cells, leads to a hypersensitivity reaction and, therefore, increased inflammation resulting in smooth muscle contraction, increased mucus secretion and oedema [1, 3].

Dupilumab is a recombinant human IgG4 (Immunoglobulin G4) monoclonal antibody that inhibits interleukin-4 and interleukin-13 signalling. IL-4 and IL-13 are major drivers of human type 2 inflammatory disease, such as atopic dermatitis, asthma, and CRSwNP. Blocking the IL-4/IL-13 pathway with dupilumab in patients decreases many of the mediators of type 2 inflammation [2, 4, 5]. A randomised controlled trial published in *The Lancet* demonstrated its efficacy in reducing polyp size and improving quality of life in patients with CRSwNP who had exhausted conventional treatment options [4]. In the United Kingdom (UK), current National Health Service (NHS) funding restricts Dupilumab to severe asthma with type 2 inflammation and moderate to severe atopic dermatitis [6]. However, in the US and EU, Dupilumab is available for use in patients with severe CRSwNP, offering a new therapeutic option for this difficult-to-treat population [2, 7].

A systematic review published in the *Cochrane Library* examined the efficacy of various biologic therapies for CRSwNP, including agents, such as tralokinumab and lebrikizumab, which specifically target interleukin-13 (IL-13) [5]. Other monoclonal antibodies, such as mepolizumab, reslizumab, and benralizumab, act against interleukin-5 (IL-5), thereby reducing eosinophil levels and associated inflammation. In addition, omalizumab, a monoclonal antibody that binds to free immunoglobulin E (IgE), interrupts the IgE-mediated inflammatory cascade by decreasing circulating IgE levels [5]. The Cochrane review focused on three biologics currently licensed for use in the UK—dupilumab, mepolizumab, and omalizumab [5, 8]. Among these, dupilumab demonstrated the greatest efficacy in improving quality of life and reducing nasal polyp burden, likely due to the larger volume of supporting evidence available [5]. Notably, the effectiveness of omalizumab is likely enhanced in patients with elevated IgE levels or underlying atopic disease, given its mechanism of action [8].

Functional endoscopic sinus surgery (FESS) is associated with an increased incidence of post-operative complications, such as mucoceles [9]. In addition, a higher number of surgeries correlates with an increased rate of disease recurrence. There is an inverse relationship between the number of surgeries and the interval between interventions; patients undergoing more procedures tend to require subsequent surgeries at shorter intervals. In Europe, biologic therapies are increasingly considered for patients with severe chronic rhinosinusitis with nasal polyps, a practice that is gaining attention in the UK as well [10]. Biologics have demonstrated efficacy in refractory CRSwNP cases, particularly when other treatment options are exhausted. However, their high cost raises questions regarding cost-effectiveness compared to surgical management [7]. The economic burden related to quality of life (QoL) impairment and lost productivity in this patient population should not be overlooked [11]. In the UK, Dupilumab is currently licensed and funded for severe asthma with an inflammatory component, with a high cost per dose [12]. Retrospective studies in both the US and the UK suggest that FESS remains the more cost-effective treatment option for CRSwNP [7, 13]. Furthermore, the optimal duration of Dupilumab therapy is not yet established, which may further increase the long-term treatment costs necessary to maintain disease remission [7, 8].

Advances in medical innovation have led to significant improvements in the prevention and treatment of many diseases. However, these developments often come with substantial costs, raising important questions about affordability, equitable access, and whether such treatments should be universally funded by the NHS [14]. High-cost therapies are subject to ongoing review by the National Institute for Health and Care Excellence (NICE). The Individual Funding Requests (IFRs) process is an alternative route for the funding of high-cost treatments not routinely commissioned in the NHS. Cases must demonstrate high clinical exceptionality and not represent a larger cohort. There needs to be a well-presented evidence base for the proposed treatment and a detailed comparison of the cost of proposed versus standard treatment, including drug costs, out-patient visits and inpatient stays that are appropriate for the case. Support from pharmacy colleagues in the preparation of an IFR request is essential. Approximately 50% of IFRs are rejected, prompting concerns about the inefficient use of clinician time and healthcare resources [14].

## Conclusion

The LIBERTY NP SINUS-24 and SINUS-52 trials, published in *The Lancet*, demonstrated that dupilumab is a safe and effective treatment for CRSwNP. In the refractory case described in this report, the patient experienced a marked improvement in both symptoms and quality of life following treatment with dupilumab. Despite its established efficacy and adoption in several European countries, dupilumab is not currently available through routine NHS commissioning in the UK. Access is limited to IFRs, which are approved only in cases deemed truly exceptional, as illustrated by the case discussed. The cost-effectiveness of dupilumab compared to surgical intervention remains under evaluation, and both financial constraints and limited access continue to be significant barriers to its widespread implementation in the UK. Nevertheless, for patients with severe, treatment-resistant CRSwNP, dupilumab and other biologics offer a promising therapeutic alternative.

## Data Availability

Patient data were used retrospectively in the manuscript.

## Abbreviations

CRS: Chronic rhinosinusitis
CRSwNP: Chronic rhinosinusitis with nasal polyps
CT: Computed tomography
ENT: Ears, nose and throat
EU: European Union
FESS: Functional endoscopic sinus surgery
IFR: Individual funding request
IgE: Immunoglobulin E
IgG4: Immunoglobulin G4
IL-4: Interleukin 4
IL-5: Interleukin 5
IL-13: Interleukin 13
NHS: National Health Service
NHSE IFR: National Health Service England Individual Funding Request
NICE: National Institute for Health and Care Excellence
NO: Nitric oxide
QoL: Quality of life
SNOT-22: Sino-Nasal Outcome Test
UK: United Kingdom
US: United States
